# Trends in neuropathology training in Sub-Saharan Africa – current curricula, resource gaps, and the potential of virtual microscopy and telemedicine

**DOI:** 10.17879/freeneuropathology-2026-9140

**Published:** 2026-01-14

**Authors:** Habiblah Jagunmolu, Emmanuel Oyetola, Kamil Ajagbe, Samuel Oyelude, Muminat Jubreel, Oluwatosin Afolayan, Dorothy Abimbola, AbdurRoqeeb Ismail, Kaothar Oyeniran, Mukhtar Ibrahim, Bright Oguntola

**Affiliations:** 1 Department of Medicine, Ladoke Akintola University of Technology, Ogbomoso (Oyo), Nigeria; 2 University Hospital Birmingham Foundation Trust, Birmingham, United Kingdom; 3 Department of Nursing, Ladoke Akintola University of Technology, Ogbomoso (Oyo), Nigeria; 4 Medical Laboratory Services, Lagos State University Teaching Hospital, Ikeja (Lagos), Nigeria

**Keywords:** Neuropathology, Sub-Saharan Africa, Diagnosis, Training, Curriculum, Virtual microscopy, Telepathology

## Abstract

**Introduction**: Neuropathology is important in the diagnosis of neurologic and neuro-oncologic diseases. But despite its immense importance, it remains underrepresented in medical training across different parts of Sub-Saharan Africa. Training in this region is limited by a low number of specialists (e.g., a ratio of 1.7 million inhabitants per an unspecialized pathologist), fragmented data, poor infrastructure, and minimal exposure. Most times, neuropathology is embedded in general pathology curricula with limited mentorship, specialized facilities, and tailored subspecialty pathways (e.g., Nigeria, Tanzania). But despite these prevailing challenges, digital tools like telepathology and virtual microscopy may help bridge those gaps.

**Aim**: This scoping review aims to understand the structure of existing neuropathology training and identify important gaps in structure and resources across Sub-Saharan Africa. It also seeks to explore how regional and global collaborations and digital innovations can be integrated to bridge these gaps.

**Methodology**: Using PRISMA-ScR guidelines, we searched literature published between 2000 and 2025 across major databases such as PubMed, Scopus, Web of Science, AJOL, and grey sources. We included and thematically analyzed studies that focused on training in neuropathology, workforce, and digital tools in Sub-Saharan Africa. We mapped out data to capture country, program type, curriculum content, resource availability, and digital tool integration. Quantitative synthesis summarized the frequency and geographic distribution of programs, while qualitative thematic analysis identified recurring patterns in training gaps, infrastructural limitations, and the application of virtual microscopy and telepathology.

**Result**: We reviewed eleven studies that indicate limited neuropathology programs, an extremely low number of neuropathologists (e.g., 0.4–0.6 per million in many Sub-Saharan Africa nations), inadequate mentorship, and limited training resources. However, telepathology and virtual microscopy show improved accuracy in diagnosis and quality training (e.g., Tanzania recorded a 35 % increment in accuracy by specialized pathologists using telepathology over general pathologists). Strengthening international collaborations also demonstrates feasibility and enhanced quality training.

**Conclusion**: Neuropathology in Sub-Saharan Africa is underdeveloped and fragmented; however, the increasing access to newer digital solutions presents low-cost options as practical alternatives for overcoming diagnostic and training obstacles. To narrow these gaps, the track toward becoming a subspecialist in neuropathology should be formed, accessible digital libraries of learning materials must be developed, and regional and international telepathology networks should be strengthened.

## 1.0 Introduction

### 1.1 Background

Neuropathology is the specialized field of neuroscience and pathology that is focused on the study and diagnosis of diseases of the brain, spinal cord, nerve and muscles through the analysis of tissue samples [1]. It serves as the cornerstone for diagnosis and the understanding of the etiology and pathogenesis of a wide variety of neurological disorders [1]. For neurodegenerative diseases such as Alzheimer’s and Parkinson’s diseases, neuropathological assessment remains the gold standard for making a definitive diagnosis because it helps in understanding the natural history of these diseases as well as in shaping their current clinical diagnostic criteria [2]. For neuro-oncology, the classification of brain tumors is also dependent on neuropathology, with increasing integration of testing for advanced molecular features in addition to the traditional microscopic examination [3].

The approach to neuropathology training varies significantly globally, usually reflecting each country’s style of medical services delivery and the level of investment in healthcare [4]. Training in high-income countries (HICs) is typically governed by a structured, highly regulated system. For instance, in the United States, neuropathology training is a two-year ACGME-accredited fellowship program after a residency training in anatomical pathology [4]. In contrast, neuropathology training in the United Kingdom and some other European countries is a recognized primary specialty program that allows medical graduates to directly undergo a 5- to 6-year training [4]. In these countries, the trainees are exposed to a large number of cases, and they benefit from digital tools and well-equipped laboratories. These systems have evolved over decades of investment in medical specialization training and represent a global standard for producing competent specialists in the field of neuropathology.

The situation in Sub-Saharan Africa is significantly different from these well-resourced settings. The region suffers from a critical scarcity of trained specialists in pathology, with pathologists-to-population estimated at less than one per 500,000 people, compared to about one per 15,000–20,000 in the United States and United Kingdom [5]. A severe lack of formal training opportunities is the direct cause of this workforce crisis. Even in places where training is in existence, neuropathology exposure is usually not adequate. For instance, an outreach-training in Ghana in 2022 discovered that there were no dedicated neuropathologists throughout the country [6]. Also, countries like Tanzania and Uganda have just 15 and 18 pathologists serving 38 million and 28 million people, respectively [7]. This lack of human resources is complicated by grossly underfunded and inadequately equipped laboratory infrastructure [8]. While infectious causes of neurological impairment (such as cerebral malaria, HIV-associated neurocognitive disorders, and meningitis) remain prevalent, there is a sharp increase in non-communicable neurological disorders driven by rapid urbanization, aging populations, and lifestyle shifts [2,7]. The rise in the cases of infectious and non-infectious neurological diseases, as well as the increased incidence of Central Nervous System tumors, emphasizes the urgent need for enhancement of neuropathology training in Sub-Saharan Africa. In addition, the infectious diseases of neurological concern are different in Africa when compared with other parts of the world hence a need for targeted training.

In the face of all these challenges, digital innovations such as virtual microscopy and telemedicine (including telepathology) have emerged. These innovations offer a way out to overcome the longstanding resource-based and geographical barriers [9]. Telepathology and virtual microscopy give trainees in resource-limited settings the opportunity to access libraries with many teaching cases, thereby overcoming the constraint of limited local case volumes and the absence of physical microscopes [9]. In addition, telepathology can link laboratories in remote areas to a global network of neuropathology subspecialty experts, leading to improved accuracy in diagnosis and cutting down turnaround times [10]. These emerging technologies give Sub-Saharan Africa an opportunity to overcome the traditional, resource-intensive stage of healthcare development by utilizing digital platforms for neuropathology education and diagnosis.

### 1.2 Rationale for the review

In spite of the important role neuropathology plays in neurological treatment, the literature on neuropathology training in Sub-Saharan Africa still remains very dispersed. There exists little published research that systematically evaluates the existing curriculum, training pathways or gaps in neuropathology training resources specific to this region. In many instances, it may be challenging to map training initiatives because they are unpublished, internal or included in broader anatomical pathology programs. Moreover, although digital pathology and telemedicine have been included in the contexts of general pathology in Africa, little is still known about their specific application in neuropathology training. Due to a lack of sufficient evidence, it is difficult to study the current status, identify gaps and create solutions specifically tailored to neuropathology training in Sub-Saharan Africa.

Therefore, the aim of this scoping review is to map the neuropathology training frameworks and curricula currently existing globally and to contextualize the findings in Sub-Saharan Africa. It also seeks to recognize major gaps in the curriculum (such as a lack of dedicated fellowship, a limited number of available slides) and infrastructure (such as inadequate neuropathology faculty, lack of digital scanners), as well as to evaluate the emerging role of telemedicine and virtual microscopy as educational equalizers. Through this, this review will create a baseline understanding that can assist in creating curriculum, developing regional partnerships and capacity-building initiatives within and beyond Sub-Saharan Africa.

### 1.3 Objectives and research questions

Objective:

To map global trends and assess neuropathology training frameworks, gaps, and emerging digital approaches in Sub-Saharan Africa.

Research Questions:

1. What neuropathology training programs exist currently in Sub-Saharan Africa?

2. What are the major curricular and resource gaps in neuropathology training in Sub-Saharan Africa?

3. How are telemedicine and virtual microscopy being used (or could be used) to improve neuropathology training in Sub-Saharan Africa?

## 2.0 Methodology

This methodology aligns rigorously with the PRISMA-ScR (Preferred Reporting Items for Systematic Reviews and Meta-Analyses Extension for Scoping Reviews) guidelines to ensure transparency and rigor. This scoping review on neuropathology subspecialty training in Sub-Saharan Africa addresses a major disparity in health distribution and resource limitations in Africa while providing evidence and highlighting the overall impact on patient care in Africa.

### 2.1 Search strategy

A comprehensive literature search was conducted from October 1st to October 4th, 2025, to access the current curricula, resource gap and extent of neuropathology in Sub-Saharan Africa, utilizing four electronic databases, encompassing both global and regional literature: PubMed, Scopus, Web of Science, and African Journals Online (AJOL). Other data were sourced from grey literature sources, including institutional curricula, policy documents, reports from the International Society of Neuropathology (ISN), and official assessments from organizations like the WHO/IARC. Search terms were combinations of: (("Neuropathology" OR "Neuro-Pathology") AND ("Training" OR "Curriculum" OR "Workforce" OR "Virtual Microscopy" OR "Telepathology")) AND ("Africa" OR "Sub-Saharan Africa").

**Figure 1: Summary of the selection process:**
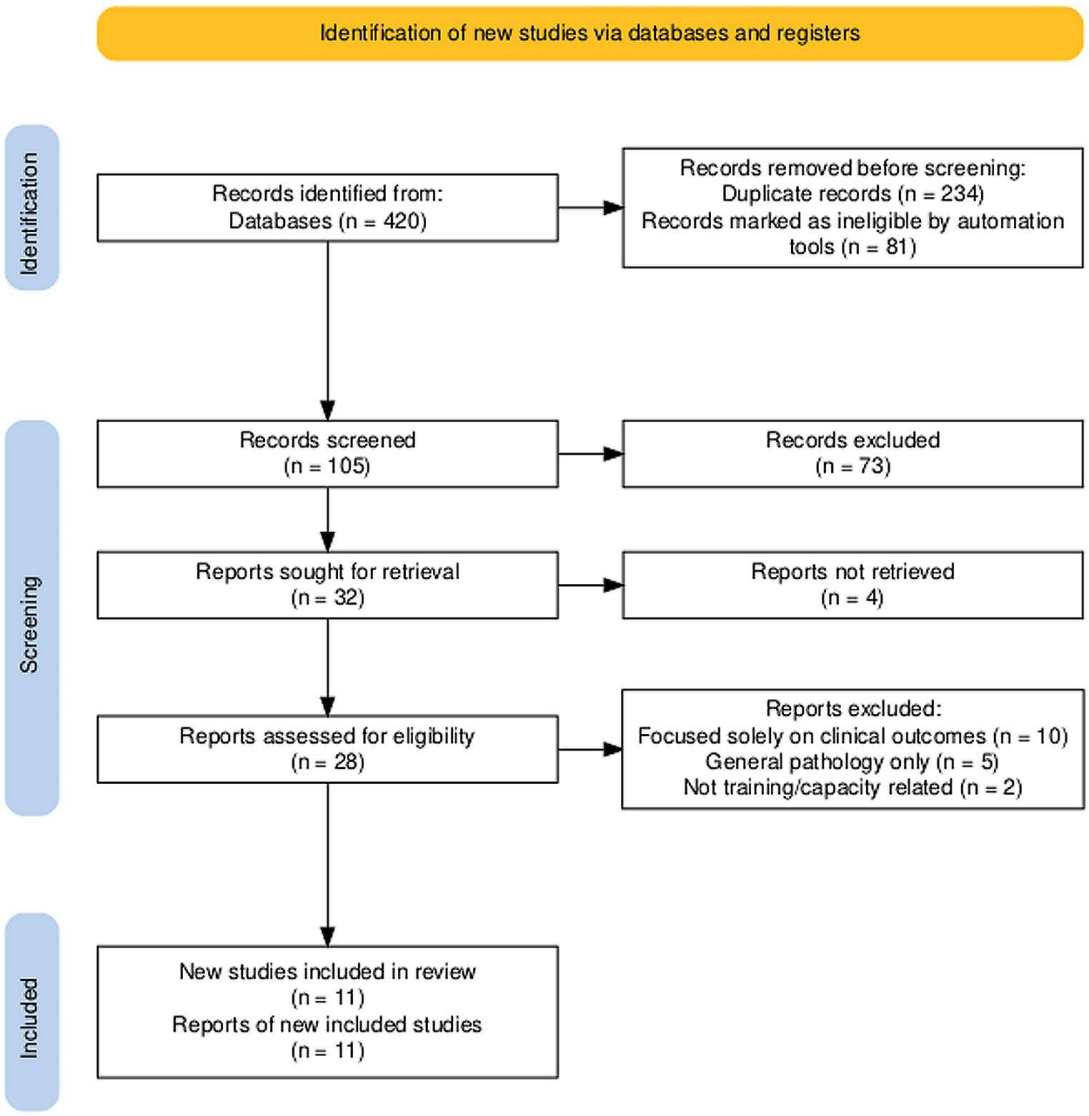


Results from each database were imported into Rayyan, where duplicates were removed, and title and abstract screening were done by two independent reviewers [H.J. and M.J.]. Discrepancies were resolved by E.O., after which full-text screening was done. Studies meeting the eligibility criteria were included in the study. See **Figure 1** for a summary of the selection process [11].

The research question is framed according to the PCC (Population, Concept, Context) framework:

- Population (P): Neuropathology trainees, general pathologists, and teaching institutions.

- Concept (C): Training Curricula, Resource Gaps, Workforce Capacity, and the potential of Virtual Microscopy (VM).

- Context (C): Sub-Saharan African countries.

### 2.2 Inclusion criteria

Studies were included if they:

- Were conducted, reported, or focused on institutions or practices in Sub-Saharan African countries.

- Reported on neuropathology specifically (training content, workforce challenges, resource capacity, or service delivery).

- Addressed at least one element of training or capacity building, including the feasibility or use of digital/virtual tools (VM, whole slide imaging (WSI), Telepathology) for education in this context.

- Were primary research studies, official institutional reports, or systematic reviews (excluding only simple editorials/opinion pieces without institutional data).

### 2.3 Exclusion criteria

Studies were excluded if they:

- Focused solely on clinical outcomes or patient management without addressing pathological capacity.

- Were conducted exclusively outside of Africa.

- Focused solely on general pathology or dermatopathology without specific mention of neuropathology capacity or curriculum.

- Were not available in English (to maintain consistent quality of data extraction).

- Grey literature sources were evaluated based on several predefined criteria to ensure their relevance and quality. Each document was assessed for its relevance to the research question, with the credibility of the source (from reputable organizations) evaluated.

### 2.4 Screening

Study selection was done using titles and abstracts, which were screened for eligibility by two independent reviewers [H.J. and M.J.]. Also, full-text articles of potentially relevant studies were assessed using predefined eligibility criteria. Discrepancies were resolved through discussion or consultation with a third reviewer [E.O.].

### 2.5 Study selection

**Table 1** shows the progression of records through the Scoping Review stages, thereby ensuring transparency and reproducibility. The review yielded 11 final documents for inclusion in the synthesis.

**Table 1:** Progression of records through the scoping review stages

**Review Stage**	**Database**	**Records Yielded (N)**	**Records Removed**	**Records Excluded**	**Exclusion Reasons**	**Included Studies (N)**
**Gender**	M	M	F	M	M	M
**Identification**	PubMed/ MEDLINE	120	0	0	-	-
**Identification**	Scopus	155	0	0	-	-
**Identification**	Web of Science	105	0	0	-	-
**Identification**	AJOL	40	0	0	-	-
**Removal**	All Databases	420	315 (Duplicates)	0	-	-
**Screening**	Title/Abstract	105	0	73	1. Not related to pathology (n=35). 2. Review/Opinion without data (n=20). 3. Not SSA focused (n=18).	-
**Eligibility**	Full-Text Retrieval	32	0	4	1. Full text unavailable (n=1). 2. Non-English (n=1). 3. Editorial/ Commentary (n=2).	-
**Final Inclusion**	Full-Text Assessed	28	0	17	1. Focused solely on clinical outcomes (n=10). 2. General pathology only (n=5). 3. Not training/capacity related (n=2).	11

### 2.6 Data extraction and synthesis

Data was extracted by two of the authors, B.O. and K.A., using a standardized form that captured study details (author, year, location, study design), primary findings on current curricula/training, key findings on resource gaps and potential of digital tool integration. The data extraction was reviewed by H.J. to ensure consistency. Findings were synthesized thematically. Key themes were analyzed, such as the impact of current training facilities on specialist density, prospect of integration of digital tool into neuropathology specialty training and global trends in neuropathology, relative to Sub-Saharan Africa.

### 2.7 Quality assessment

Quality of included studies was assessed using the Critical Appraisal Skills Programme (CASP). Two authors (H.J. and M.J.) independently assessed each article for risk of bias and strength of evidence. Disagreements were resolved through discussion and the intervention of E.O. **Table 2** shows detailed information.

**Table 2:** Quality assessment for included studies.

**CASP-Adapted Quality Domain**	**El Jiar (Telepath.)**	**Razzano (Capacity)**	**Zerd (Feasibility)**	**Folaranmi (** **Social Media** **)**	**Chan (Neuro-Onc)**	**ISN (Workshop Report)**	**Kalaria (Symposium)**	**Olatunji (3D Print)**	**WHO/IARC (Policy)**	**Olasode (Practice Review)**	**Udoh (Case Series)**
(Q1) Was there a clear statement of the aims/objectives?	Y	Y	Y	Y	Y	Y	Y	Y	Y	Y	Y
(Q2) Was the design appropriate to address the aims?	Y	CT	Y	CT	Y	CT	CT	Y	Y	CT	Y
(Q3) Was the data collection method clearly described/justified?	Y	CT	Y	Y	CT	N	CT	N	Y	N	Y
(Q4) Was the setting and context clearly described as SSA?	Y	Y	Y	Y	Y	Y	Y	Y	Y	Y	Y
(Q5) Were the limitations or biases adequately discussed?	Y	Y	Y	Y	Y	N	Y	Y	CT	Y	CT
(Q6) Is there a clear statement of findings/recommendations?	Y	Y	Y	Y	Y	Y	Y	Y	Y	Y	Y

Y = YES, N= NO, CT= Cannot tell.

## 3.0 Results

### 3.1 Overview of included studies

This study utilizes 11 studies, all published between 2015 and 2024, focused primarily on neuropathology training and resource challenges in Sub-Saharan Africa. The study designs employed includes bibliometric analyses, literature reviews, perspective articles, descriptive and retrospective cohort studies, case series and grey literature workshop reports, which provides a comprehensive picture of the neuropathology training across Sub-Saharan Africa [12]. Geographically, the literature is concentrated in Sub-Sahara Africa countries like Nigeria, Tanzania, Kenya and broader Pan-Africa analysis. There is an increase in publication post 2020 which reflects a growing attention to neuropathology training innovations and challenges in the region. The included studies showed a limited and scattered neuropathology training framework. The studies documented significant shortage of subspecialty neuropathologists, deficiency in infrastructures (such as deficit in staining tools) and autopsy findings, which further complicate the problem [12]. See **Table 3** for detailed information.

**Table 3:** Included studies

**Author(s) an** **d year**	**Country/ Region ** **foc** **us**	**Study ** **des** **ign/ ** **do** **cument ** **type**	**Primary ** **findings on current curriculum/training**	**Key ** **findings on resource gaps/service**	**Digital Tool (VM/Telepathology) Integration & Potential**
El Jiar M. et al. (2024) [12]	Africa	Bibliometric Analysis and Literature Review	This study confirms the existence of fragmented training efforts in Sub-Saharan Africa, coupled with low Neuropathology specialist density.	The number of publications on telepathology in Africa is low compared to other bibliometric studies; Challenges faced by telepathology include: implementation challenges power supply, and hardware costs.	Establishes significant interest in Telepathology/WSI (whole slide imaging). Study confirms technical feasibility in Africa following proper policy and funding.
Razzano D. et al. (2022) [13]	Sub-Saharan Africa	Perspective Review	Emphasizes the need for structured training programs in order to utilize digital solutions in pathologic diagnosis.	Challenges include: limited infrastructure, poor systemic implementation, and lack of sustainability and pathologist engagement.	Surge in potential for capacity building and access to specialty consultation. Improved specialty training skills and global standard. Enhanced quality control. Improved clinical trial implementation. Improved patient care.
Zerd F. et al. (2020) [14]	Tanzania	Case Series	Study highlights limited local training capacity and reliance on external expertise. Annotates improvement in diagnostic accuracy from 36 % by general pathologists to 71 % by a neuropathologist using static telepathology (or from 76 % to 88 % with less stringent criteria)	Limitations include: limited technical infrastructure hampers neuropathologic training with subsequent affectation of neuropathologic diagnosis. There were 35 pathologists serving 58 million people in Tanzania (a ratio of 1.7 million inhabitants per pathologist), none of whom are subspecialty trained in neuropathology.	This study confirms successful tele-neuropathology consultation using basic photomicrography in some regions in Sub-Saharan Africa. There is a need for more implementation policies to ensure widespread feasibility of this type of consultative relationship in surgical neuropathology.
Folaranmi O.O. et al. (2022) [15]	Nigeria	Descriptive Study	Explores the use of virtual environments (e.g., social media) for pathology education. Social media sites serve as sources of readily accessible, free, high-quality information to pathologists and trainees through academic discussions, quizzes, journal clubs, and informal consult.	Addresses digital literacy and willingness to participate in virtual learning.	Relevant to assessing the general digital acceptance and literacy required for VM.
Chan M.H. et al. (2015) [16]	Developing World	Literature Review	Identifies insufficient training and lack of proper tissue handling expertise as a major challenge in pediatric brain tumor management. Buttresses that there is no internationally agreed upon or universal standard as to what training qualifies one to be a neuropathologist.	Uganda has 0.64 pathologists per million citizens, and Tanzania has 0.39 pathologists per million. Neuropathology depends on government funding which is often unreliable. Many developing nations do not require death certificates to be filled out by trained medical personnel, and many deaths go unreported.	Supports the need for standardized and centralized virtual case repositories in order to compensate for low local volume.
ISN Report (2024) [17]	Kenya	Workshop Report (Grey Literature)	Details the specific curriculum content (e.g., Neurodegeneration, Stroke) delivered in outreach workshops.	Neuropathologic training is non-existent due to scarcity of mentors/supervisors.	Directly correlates the use of Digital Slides (VM) by international faculty during training events with improved training quality.
Kalaria R. et al. (2024) [18]	LMICs	Symposium Report	Highlights that resources severely limit diagnosis and research capacity for neurodegenerative diseases.	Major diagnostic gap for neurodegenerative diseases is due to limited number of Neuropathologists and autopsy facilities. Researchers needing neuropathology services face the following challenges: outdated traditional teaching infrastructure.	Digital platforms are crucial tools for international research collaboration in neuropathology.
Olatunji G. et al. (2023) [19]	Africa	Literature Review	Embraces the recent shift toward digital-age education and highlights that it is an essential tool for medical schools in Africa. Modernizing medical education especially via 3D printing application has been shown to improve training in anatomic pathology.	Outdated traditional teaching infrastructure.	This study contextualizes the general willingness and feasibility of integrating virtual tools like VM into medical education, suggesting a positive impact on learning and training outcome.
WHO/IARC (2021) [20]	Nigeria	Policy Document	Highlights that neuropathology content is often embodied and marginalized within general pathology residency, implying that no formal subspecialty pathways exist in most tertiary teaching hospitals in Nigeria.	Lack of essential laboratory equipment. National gaps in immunohistochemistry facilities and cryostat access.	Critical infrastructural barriers must be overcome in order to ensure effective VM deployment and incorporation.
Olasode BJ et al. (2016) [21]	Nigeria	Retrospective Cohort	Scarcity of local mentorship. Profound challenges in subspecializing in neuropathology. Lack of modern tools to compete with international standard.	This study confirms the constant need for telediagnosis to ensure quality assurance and consultation.	Supports the use of digital tools as a necessary consultation bridge due to specialist shortage.
Udoh M.O. (2022) [22]	Nigeria	Case Series	High local burden of CNS infections and tumors, confirming the necessity of specialized skills. Lack of adequate training, facility and mentorship for professionals willing to subspecialize.	Quantifies the high case volume that must be used for effective neuropathology training.	The high burden of cases confirms the clinical necessity for scalable digital teaching resources and specialty training.

### 3.2 Neuropathology training landscape

The neuropathology training in Sub-Saharan African is characterized by limited structure and dependence on external expertise due to insufficient local training capacities. Zerd et al. (2020) and Olasode et al. (2016) highlighted a fragmented training environment with little specialized rotations and inadequate recognition for subspecialties [20]. There is lack of standardization in the training curriculum and duration, with episodic training being reported due to shortage of teachers, mentors and supervisors [17]. In Sub-Saharan Africa, no country offers a standalone, board-certified neuropathology residency program; however, formal Anatomic Pathology training (leading to MMed or College Fellowships) exists in roughly 15–20 % of nations, including South Africa, Nigeria, Kenya, Tanzania, Ethiopia, and Ghana [5,21]. Within these programs, neuropathology is integrated as a brief, "fragmented" module (often 4–8 weeks) focused on basic H&E staining of surgical specimens and neuro-infections, with geographic differences defined by a tiered spectrum of development [14]. Southern Africa (most importantly, South Africa) represents the most advanced tier with sub-specialty registration, molecular diagnostics, and brain banks, whereas East Africa (e.g., Kenya, Tanzania) and West Africa (e.g., Nigeria) rely on case-dependent apprenticeship models and virtual collaborations to bridge the gap in sub-specialized faculty and infrastructure [5,14,21]. Due to increased burden of central nervous system diseases, there is clinical demand for neuropathologists in Sub-Saharan Africa countries, yet the local training programs are still struggling to keep pace with the high demand [22]. There are inconsistent or weak assessment frameworks and training duration and curriculum varies widely with no standardization across institutions. The neuropathology content in Sub-Saharan Africa is often limited to a few topics such as neurodegeneration and cerebrovascular diseases, without any comprehensive coverage of the neuropathological spectrum [17]. This contrasts with the global standards where neuropathology training has well-structured subspecialty rotations, rigorous assessments and competency-based progression [16]. Sub-Saharan Africa faces major challenges in replicating the global standards due to shortages in specialized personnel, lack of infrastructures and funding. In countries like Tanzania, the neuropathologist to population ratio is low, impeding effective mentorship and supervision [14]. Training in Sub-Saharan Africa is also heavily reliant on external expertise and international collaborations, which, while beneficial, are not always sustainable and episodic [21]. There is an increased recognition of digital modalities, such as virtual microscopy and telepathology to supplement the traditional training method and to also overcome resource limitations. These platforms have proven beneficial in exposing trainees to more and broader case volumes and improving diagnostics skills. Although integration is still inconsistent and rely on the infrastructure capacity [12,15].

### 3.3 Resource and infrastructure gaps

Resources and infrastructure limitations are major barriers to effective neuropathology training and service delivery in Sub-Saharan Africa. There are widespread laboratory deficits such as insufficient histology units, lack of proper staining tools, limited autopsy services that are essential for neuropathological training and diagnosis [16,20]. Many institutions still lack access to immunohistochemistry facilities and cryostats, which are important for advanced neuropathological analysis. This scarcity of equipment hinders both the training quality and the ability to conduct accurate specialized diagnoses [20]. Human resources constraints further exacerbate these challenges. The density of neuropathologists still remains critically low, with some countries having less than a pathologist per million people and even fewer with subspecialty training in neuropathology [14,16]. The scarcity of experienced neuropathologists limits mentorship opportunities for training which creates a cascading effect where trainees receive minimal support and supervision [17,21]. Funding and institutional barriers also restrict progress. Most neuropathology training centers rely heavily on governments that are unpredictable and external funding, which is irregular and inadequate [16,21]. Institutions may not prioritize neuropathology due to its perceived niche status, which results in limited budget and allocation for essential infrastructures and development. Poor supply chains and maintenance support are other systemic issues that also affects the sustainability of available equipment [18].

### 3.4 Integration of virtual microscopy and telemedicine

There is increasing evidence supporting the use of virtual microscopy (VM) and telepathology in Sub-Saharan Africa as valuable tools in neuropathology training and diagnosis. Several studies have demonstrated the successful implementation of tele-neuropathology and digital pathology collaborations. This highlights their potential to overcome geographical and resource constraints in the region [12,14]. Zerd et al. (2020) reported an improved diagnostic accuracy in Tanzania through the use of static telepathology consultations, with accuracy increasing from 35 % by general pathologist to 71 % by neuropathologist through online consults. El Jair et al. (2024) also affirmed the feasibility of whole slide imaging and telepathy in Africa, conditioned on adequate funding and supportive policies. The International Society of Neuropathology (ISN) workshops incorporate the use of digital slides by international faculty, enhancing the quality of training despite limited local resources (ISN, 2024). Folaranmi et al. (2022) also discussed the use of social media as an informal virtual environment for training by providing access to academic discussions, journal clubs, quizzes, and consultation opportunities for trainees that extend beyond the traditional classroom setting. These tools offer a scalable platform to increase trainee exposure to broad and diverse cases and expert mentorship. Despite these successes, there are still barriers that impede the widespread implementation of virtual microscopy (VM) and telepathology. Cost is still a major challenge, with high investments required for scanning equipment, servers and reliable internet infrastructure [12]. Limitation in power supply further complicates consistent, high quality digital slide transmission [13]. Varying technical skills among institutions and trainees also poses challenges, which necessitates investment in digital literacy and training to ensure effective technology adoption [15]. In spite of these barriers, the success of international collaborations demonstrates the feasibility of building telepathology networks that can be institutionalized to improve both training and clinical diagnosis across Sub-Saharan Africa.

### 3.5 Emerging global models

International neuropathy training collaborations offer valuable insights and models that can be adopted to improve training in Sub-Saharan Africa. Several initiatives showed the benefits of shared digital slide libraries, exchange fellowship programs, and collaborative workshops as effective strategies to address expertise gaps in low resource areas [12,17]. These models leverage technology and global partnership to compensate for the deficiencies in local training and improve access to specialized knowledge. A successful example is the use of a shared digital slide repository, that allows trainees to review diverse and complex neuropathology cases remotely. This approach not only standardizes learning but also broadens case exposure [16]. The creation of international digital libraries ensures there is continuous availability of training materials and expert contributions.

Exchange fellowship programs represent another promising model, where trainees from Sub-Saharan Africa receive hands-on experience and training in high-income country (HIC) institutions. These programs will provide immersive experience in world class laboratories, with structured curricula and mentorship, exposing the trainee to advanced diagnostic methods and best practices [13]. Collaborative workshops and symposia organized by international bodies like International Society of Neuropathy (ISN) have also proven effective. These events enable direct knowledge transfer through interactive sessions, digital slide demonstrations and shared expertise, providing focused training opportunities for low resource settings [17,18].

### 3.6 Challenges identified

Several critical challenges hinder the advancement of neuropathology training and delivery in Sub-Saharan Africa. There are limited awareness and prioritization of neuropathology as an essential and distinct subspecialty within the broader healthcare and academic system. Neuropathology remains marginalized and subsumed under general pathology without dedicated resources in many institutions [20]. There are also fragmented data and scarce funding, which further complicate the problem. Comprehensive data on neuropathology capacity, training outcomes and diseases burden remain limited and inconsistent in many regions, which limits evidence-based planning and advocacy [18,21]. Low resources and funds dedicated to neuropathology research and education specifically further impedes the ability to implement sustainable programs, invest in infrastructures or conduct local studies that could help develop curriculum and service needs [16]. Technology adoption, although promising, faces significant barriers related to cost, infrastructures and sustainability. High initial capital expenditures for virtual microscopy equipment, unreliable internet connectivity, epileptic power supply and limited technical expertise challenge the scaling of digital pathology solutions [12,13].

## 4.0 Discussion

This scoping review sought to evaluate current curricula, resource gaps, and the potential of virtual microscopy (VM) and telemedicine in neuropathology training across Sub-Saharan Africa. A number of recurring patterns emerge: fragmented training efforts, extremely low specialist density, major infrastructural barriers, and an encouraging but under-exploited potential for digital tools. For instance, El Jiar and his colleagues documented a relatively low number of telepathology publications from Africa, underscoring both the underdevelopment of the field and the sparse engagement in digital pathology research in the continent [12]. This low publication output reflects underlying structural constraints: few neuropathology specialists, lack of formal subspecialty pathways, and dependence on episodic external interventions. Available evidence indicates that neuropathology training across Sub-Saharan Africa remains limited and poorly structured. In many countries, there are no established subspecialty pathways in neuropathology, and most residency programs only cover it as a small component within general pathology training rather than as a distinct module or fellowship [17]. A striking empirical example of digital intervention comes from Tanzania: in a static-image teleneuropathology study, diagnostic concordance rose from 36 % (via general pathologists alone) to 71 % (strict criteria) or 88 % (less stringent criteria) when an expert neuropathologist reviewed remotely [14]. That jump is not trivial: it offers a concrete demonstration that digital methods can substantially elevate diagnostic accuracy under constrained local conditions. But the baseline disparity – few locally trained neuropathologists, limited subspecialization opportunities – is itself symptomatic of deeper systemic neglect. Indeed, some studies report that a country may have only a handful (or none) of neuropathologists serving millions of people. Viewed globally, the gap is stark. In wealthier settings, neuropathology fellowships, advanced subspecialty training, readily available digital slide systems, and institutional support are normative. In contrast, Sub-Saharan Africa remains in a catch-up mode, trying to build fundamental capacity. The telepathology adoption patterns in Sub-Saharan Africa contrast with trajectories in high-income settings, where whole slide imaging (WSI) and dynamic telepathology are increasingly standard. Yet, amidst the deficits, there lies transformative potential in virtual microscopy (VM) and telemedicine for leveling the field in pathology education.

There are significant shortages in infrastructure and resources. Inadequate laboratory facilities (such as immunohistochemistry and cryostat access), irregular power supplies, constrained bandwidth, expensive hardware, and a lack of local mentorship and subspecialists are some of the major obstacles noted. For example, a bibliometric analysis found that although telepathology/whole slide imaging (WSI) is becoming more popular, implementation in Africa is still limited by cost and power supply [12]. Similarly, a literature review by Chan and colleagues emphasizes that many developing nations simply lack death certification by trained medical personnel, histopathology volume is high, but training is insufficient for neuropathological practice. These systemic deficits undermine both training and service delivery [16]. Despite such challenges, the potential of VM and telemedicine in neuropathology training is compelling. Across several studies, digital approaches (e.g., static telepathology, whole-slide imaging) improved diagnostic accuracy and opened specialist consultation avenues. There was an improvement in diagnostic accuracy from 36 % to 71 % using static telepathology by non-specialist pathologists. This suggests that digital tools can act as force-multipliers in low-resource contexts: improving training opportunities, facilitating remote consultation, and accelerating capacity development. Furthermore, many trainees in Sub-Saharan Africa report access to smartphones and internet usage, pointing to a baseline digital literacy that could support VM/telepathology adoption [14]. In this way, VM/telepathology offer a transformative pathway toward more equitable neuropathology education and service. That said, it is essential to recognize that the digital potential will only be realized if the systemic barriers are addressed. The mere availability of VM slides or teleconsultation links cannot substitute for sustainably financed infrastructure, qualified mentors, pathology-service integration, and policy frameworks that embed digital training within curricula. Without addressing issues such as local pathologist retention, maintenance of digital systems, power outages, and internet reliability, digital tools risk being a “nice add-on” rather than a core component of training. In sum, the major patterns across Sub-Saharan Africa are: minimal formal neuropathology curricula, severe infrastructure and mentorship gaps, but a valuable emerging opportunity afforded by digital tools. When contrasted with global standards, the scale of the deficit becomes even more apparent. The transformative potential of VM/telepathology is real but contingent on systemic enablers.

The incorporation of structured neuropathology modules into postgraduate curricula should be a top priority for Sub-Saharan Africa governments and academic institutions in order to address these issues. These initiatives would guarantee that students are exposed to fundamental skills in immunohistochemistry, molecular diagnostics, histomorphology, and autopsy neuropathology. Partnerships at the regional and global levels should concentrate on creating telepathology hubs and mentorship networks that link students with professionals, allowing for ongoing education and consultation despite distance [13,17]. It is important to note that a primary limitation of this review is the reliance on indexed medical databases, which may omit relevant “grey literature” such as institutional curricula or internal regional reports. While we mitigated this by manually searching the websites of African medical colleges and following citation chains, the potential for missing non-formalized or locally published data remains. At the time of writing, Del Bigio et al. 2014 Neuropathology Training Worldwide – Evolution and Comparisons, we searched for information about neuropathology training and practice in Africa and could find very little except for small mention of neuropathology in the documents for Fellowship of the College of Pathologists of South Africa in Anatomical Pathology. It seems, not much has changed. The current blueprint for FC Path(SA) Anat Examination mentions “Peripheral nerve and skeletal muscle; The central nervous system; The eye” as part of the curriculum [23].

## 5.0 Recommendations

Neuropathology training in Sub-Saharan Africa remains underdeveloped due to incompetent curricula, inefficient human resources, and persistent infrastructural deficits. Addressing this critical neurological workforce deficiency requires radical reform in education, especially in neuroanatomy, mastery of which is essential for fostering student interest and developing the necessary specialized workforce [24]. Current educational structures in Sub-Saharan Africa, exemplified by studies in Cameroon, face numerous intrinsic and extrinsic challenges that actively contribute to “neurophobia”, the pronounced fear of neural sciences among medical students [24].

A primary area for reform is the teaching paradigm, which currently relies predominantly on outdated didactic methods, such as PowerPoint lectures (used by 83.2 % of faculty in Cameroon) [24]. This approach is perceived to limit students’ understanding, as PowerPoint slides may not adequately convey the true complexity of neuroanatomy or facilitate the appreciation of spatial reasoning skills necessary for mastery [24]. Furthermore, a significant extrinsic factor contributing to learning difficulty is the lack of allocated instructional time; 85.8 % of students in one survey reported that the time dedicated to neuroanatomy teaching was inadequate. This issue is aggravated by the scarce and uneven distribution of specialized neurological faculty (such as neurosurgeons and neurologists) [24]. Consequently, teaching is often handled by non-specialists, which may hinder students' grasp of clinical relevance and exacerbate neurophobia. Perhaps the most pressing resource deficit is the critical underutilization of hands-on learning, as cadaver dissection is used in only a few cases. The scarcity of cadaveric laboratories is generally due to pervasive funding, ethical, cultural, and infrastructure barriers across parts of Africa.

The potential of virtual microscopy (VM) and simulation technologies is immense for neuropathology training. VM offers significant advantages over traditional light microscopy in general pathology education, including increased flexibility, efficiency, standardization, and cost-effectiveness [25]. It has been shown to enhance student engagement and collaboration and results in satisfactory, or sometimes boosted, academic performance [25]. The feasibility and scalability of digital delivery for complex specialties have been proven through successful pilot projects implementing online postgraduate training programs, such as hematopathology, for trainee pathologists across Kenya and Zambia, demonstrating wide geographical uptake and interaction via platforms like Canvas and Zoom [26]. For neuroanatomy specifically, simulation tools, including augmented reality (AR) and virtual reality (VR) models, are highly recommended for resource-limited settings because they effectively facilitate the appreciation of 3D relationships and spatial reasoning skills, critical skills otherwise gained through scarce cadaveric experience. Most medical students have been found to recommend the usage of neurostimulation practical to improve neuroanatomy teaching. Ultimately, sustainable transformation requires combined efforts between medical institutions, policymakers, and international partners to facilitate curriculum reform and the incorporation of cost-effective simulation tools [27].

Establishment of a regional College of Neuropathologists for Africa, modeled on COPESCA's success, will harmonize curricula, training programs and examinations for neuropathologist, addressing the scarcity of specialists amid few existing programs and brain disorder burdens. Drawing from COPECSA's model, which since 2010 trained 120 fellows, secured grants, and ran workshops – such a college could partner with international organizations for accreditation, mentorship, and advocacy to elevate safe neuropathology practice [28].

## 6.0 Conclusion

Neuropathology training in Sub-Saharan Africa remains underdeveloped with major gaps in human capacity and infrastructure [13]. As with several populated countries, most consider neuropathology a subspeciality and do not formally recognize its valve hence causing a lack of structured curricula [4]. Countries with standard neuropathology training have long histories of neuropathology research, greater wealth and expenditures on health services [4]. Lack of essential structures and equipment needed for timely and accurate diagnosis also puts the training in jeopardy [13]. The era of digital pathology compensates for the limited number of specialists and offers essential tools needed for clinical work [10]. The use of virtual microscopy provides additional tools that help to link whole slide images with existing laboratory information systems, correlate pathological data, and test result data available per patient [10]. This offers the ability to perform complex image analysis and aid routine diagnosis [10]. The development of this form of digital pathology in low resource countries has to overcome some factors; high cost of acquisition and maintenance with each country's per capita expenditure being a major factor is to be considered [13]. Power supply and high-speed internet are two major poles of infrastructure needed for telepathology which is lacking in African countries [29]. The digital slides are of high resolution and require large internet service for function and with many remote laboratories in Sub-Saharan Africa lacking internet access, this can be a limitation [29]. The government and hospital management have huge roles to play by making investments in the development of required infrastructure and creating a business environment for telecommunication companies to function [29]. Collaborations allow for scientific opportunities between Africa and other developed countries utilizing the benefits of digital pathology for diagnosis, teaching and research [29]. Creating a structured curriculum for neuropathology training and providing needed infrastructure for digital pathology requires intervention from the government, joint collaborations and development of research programs.

## Author contribution

H.J. conceptualized the study and did an initial literature search. Article screening for eligibility and quality assessment was done independently by H.J. and M.J., with E.O. resolving discrepancies and conflict through consultation. B.O. and K.A. extracted the data from included articles. Data extraction was reviewed by H.J., S.O., and O.A. drafted the result and discussion section respectively. A.I., M.I., K.O. and D.A. ensured data organization and critical revision of the manuscript for intellectual content. H.J. and E.O. were involved in supervision and final review. All authors read and approved the final manuscript.

## AI use disclosure

The manuscript text was refined with AI assistance (Gemini) for improving grammar, clarity, and tone. The authors confirm that the AI tool was used solely for editorial refinement and did not contribute to the conceptualization, data analysis, writing or formation of the conclusions, which remain the sole responsibility of the authors.

## Conflict of interest statement

The authors declare no conflict of interest.
